# The effects of natural selection across molecular pathways in *Drosophila melanogaster*

**DOI:** 10.1186/s12862-015-0472-4

**Published:** 2015-09-21

**Authors:** Jeffrey P. Vedanayagam, Daniel Garrigan

**Affiliations:** Department of Biology, University of Rochester, Rochester, New York, 14627 USA

**Keywords:** Adaptation, *Drosophila melanogaster*, Pleiotropy, RNA interference

## Abstract

**Background:**

Whole-genome RNA interference post-transcriptional silencing (RNAi) is a widely used method for studying the phenotypic effects of knocking down individual genes. In this study, we use a population genomic approach to characterize the rate of evolution for proteins affecting 26 RNAi knockdown phenotypes in *Drosophila melanogaster*.

**Results:**

We find that only two of the 26 RNAi knockdown phenotypes are enriched for rapidly evolving proteins: innate immunity and regulation of Hedgehog signaling. Among all genes associated with an RNAi knockdown phenotype, we note examples in which the adaptively evolving proteins play a well-defined role in a given molecular pathway. However, most adaptively evolving proteins are found to perform more general cellular functions. When RNAi phenotypes are grouped into categories according to cellular function, we find that genes involved in the greatest number of phenotypic categories are also significantly more likely to have a history of rapid protein evolution.

**Conclusions:**

We show that genes that have been demonstrated to have a measurable effect on multiple molecular phenotypes show higher rates of protein evolution than genes having an effect on a single category of phenotype. Defining pleiotropy in this way yields very different results than previous studies that define pleiotropy by the number of physical interactions, which show highly connected proteins tend to evolve more slowly than lowly connected proteins. We suggest that a high degree of pleiotropy may increase the likelihood of compensatory substitution, consistent with modern theoretical work on adaptation.

**Electronic supplementary material:**

The online version of this article (doi:10.1186/s12862-015-0472-4) contains supplementary material, which is available to authorized users.

## Background

The visibility of a protein to natural selection depends upon the phenotypic consequences of mutations to its regulatory and structural sequences. For most proteins, the phenotypic consequences of mutations first manifest at the cellular level, specifically with respect to the protein’s ability to participate in a suite of molecular interactions. This context proximally determines both the level of sequence constraint and how often a protein produces evolutionary adaptations. For over forty years, biologists have endeavored to identify variables that predict the rate of protein evolution [1, 2]. Proteome-level statistical analyses generally find that expression pattern, breadth of interactions, and the genomic context of coding sequences are all correlated with the rate of protein evolution [3]. Even the position of proteins in molecular interaction pathways (upstream or downstream) accounts for some variance in evolutionary rate [4]. It is also widely appreciated that molecular pathways involved in immunity or genome defense are often enriched for adaptively evolving proteins [5, 6]. As functional and genomic data continue to accumulate, the tools are now available to address in detail whether certain categories of pathways are more or less impacted by natural selection.

The targeted knockdown of individual genes with short interfering RNA molecules (RNAi) is routinely used to assay the relative effect of proteins on a measurable phenotype of interest [7]. While the phenotypic effects of gene knockdown are not necessarily representative of the effects of all possible point mutations [8], they are indicative of the relative importance of the protein in different molecular pathways. This study presents an evolutionary analysis of proteins found to have significant knockdown effects in 26 whole-genome RNAi experiments in *Drosophila melanogaster*. We ask whether groups of genes affecting a given phenotype are preferentially subject to positive natural selection, relative to a random sample from the genome. Furthermore, we identify which of these genes are most impacted by recurrent positive selection. The results indicate that both immunity and cell signaling pathways are enriched for rapidly evolving proteins and that proteins with wider pleiotropic effects are more rapidly evolving than proteins that affect a narrower range of phenotypes.

## Results and discussion

### Natural selection across pathways

Each RNAi experiment *k* yields a set of *n*_*k*_ genes that, upon knockdown, cause a significant measurable change to the phenotype. The threshold for statistical significance of the magnitude of change is standardized across studies. Then, using population genomic data from *D. melanogaster* and two outgroup genomes, for each phenotype, we estimate the direction of selection statistic (DoS), which is defined as the difference between the proportion of substitutions and polymorphisms that are nonsynonymous. Under strictly neutral evolution, DoS is expected to be zero, and it is positive when the proportion of substitutions that are nonsynonymous is higher than the proportion of polymorphisms that are nonsynonymous, indicative of positive natural selection. Alternatively, DoS is negative when the proportion of polymorphisms that are nonsynonymous is higher than the proportion of substitutions that are nonsynonymous, suggestive of weak negative selection [9]. For each set of *n*_*k*_ genes influencing a phenotype, we determine the average DoS for all genes associated with that phenotype, and using a two-tailed randomization test, we further determine whether an RNAi phenotype is enriched for genes subject to positive natural selection, compared to a random sample from the genome. We note that this test is designed to detect lineage-specific recurrent natural selection.

The average number of genes significantly influencing a single RNAi knockdown phenotype is 40 and the number ranges from only five for the “cell size regulation” phenotype to 113 for the “RTK-Ras-ERK signaling decrease” phenotype (Table 1). We find two RNAi knockdown phenotypes are affected by groups of proteins with a significantly elevated mean proportion of amino acid substitutions. One of these phenotypes involves “innate immunity”: 10 out of 13 genes involved in this RNAi knockdown phenotype have positive DoS values ranging from 0.015–0.333. Of these, four genes *imd*, *Dredd*, *ird5* and *Relish* act upstream in the immune deficiency (IMD) pathway, which activates the NF- *κ*B cascade to produce antimicrobial peptides as a defense response against microbial pathogens [10]. The other phenotype with genes showing a significantly elevated mean proportion of amino acid substitutions is the “hedgehog signaling decrease” phenotype. Hedgehog (Hh) signaling is a conserved cell-signaling pathway in animals, which mediates embryonic development and tissue homeostasis [11]. Of the 25 genes that have positive DoS values in the Hh signaling decrease phenotype, only two genes, Cubitus interruptus (*Ci*) and Fused (*fu*), play a specific well-characterized role in the Hh signaling complex, in particular, both act upstream in the signaling cascade. The Ci protein is a zinc finger domain encoding transcription factor, which controls the transcription of Hh target genes [12]. The Fused protein is a kinase, which forms a protein complex with Ci, and another upstream Hh pathway protein Smoothened (*Smo*) to regulate the downstream Hh target genes [13]. It is interesting to note that in both phenotypes, we find genes with positive DoS values play crucial upstream roles. This finding is in agreement with protein interaction data from *Saccharomyces cerevisiae*, and *D. melanogaster*, in which the upstream genes in molecular networks tend to show signatures of rapid evolution, while the downstream genes tend to be more conserved [4, 14]. Of the 26 RNAi knockdown phenotypes, 16 are related to either intracellular signal transduction or involved in cell surface receptor signaling. If rapidly evolving genes randomly associate with RNAi phenotypes, it is not unexpected to observe 1 of 16 cell signaling pathways being enriched, however previous studies of protein evolution in *D. melanogaster*, that rely on gene ontology annotation, do not identify any cell signaling pathway as enriched for rapidly evolving genes [15].

**Table 1:** The 26 RNAi knockdown phenotypes surveyed in this study. Bolded lines indicate phenotypes that are enriched for proteins that significantly deviate from the genome average in their direction of selection (DoS) statistic

Phenotype category	RNAi knockdown phenotype	*n* ^a^	DoS
Regulation of intracellular signal transduction	Akt-TOR signaling decrease	24	–0.019
	Akt-TOR signaling increase	22	–0.118
	Hippo signaling decrease	99	–0.005
	Hippo signaling increase	54	–0.021
	JAK/STAT signaling decrease	10	0.044
	JAK/STAT signaling increase	6	–0.075
	RTK-Ras-ERK signaling decrease	113	–0.045
	RTK-Ras-ERK signaling increase	55	–0.018
Cell surface receptor signaling pathway	**Hedgehog signaling decrease**	**48**	**0.043** ^b^
	Hedgehog signaling increase	30	0.006
	Notch signaling decrease	16	–0.018
	Notch signaling increase	18	–0.107
	Toll signaling decrease	17	0.028
	Toll signaling increase	11	0.005
	Wnt signaling activity	90	–0.043
Regulation of transposon integration	*Blood* TE activity increase	43	0.011
	*Burdock* TE activity increase	15	–0.080
	*HeTA* TE activity increase	38	0.024
	*TAHRE* TE activity increase	41	–0.002
Innate immune response	Influenza replication decrease	18	–0.047
	**Innate immunity**	**13**	**0.097** ^b^
	*M. fortuitum* infection decrease	27	–0.111
Regulation of extent of cell growth	Cell size regulation	5	0.012
	Cell growth and viability	96	–0.014
Regulation of circadian rhythm	CRY degradation	93	0.006
Hypoxia-inducible factor-1alpha signaling pathway	Hypoxia induced transcription	49	–0.025
N/A	Lethals	2853	–0.031
N/A	Whole-genome	11148	–0.035

^a^Number of genes significantly affecting the RNAi knockdown phenotype

^b^Value is significantly greater than a random sample from the genome

### The role of positively selected proteins

Across all 26 RNAi knockdown phenotypes, there are a total of 11 genes encoding proteins with significantly elevated numbers of adaptive amino acid substitutions, identified from the McDonald-Kreitman (MK) test (Table 2). Several of these proteins are considered “conserved” components of cell signaling pathways. For example, *Sik3* encodes a kinase in the core Hippo pathway [16] that functions as a negative modulator of Hippo signaling. Additionally, other proteins that specifically affect Hippo pathway activity also experience recurrent positive selection, as is the case for the H3-K36 methyltransferase gene *Set2*, which has previously been characterized as a member of an evolutionarily conserved family of histone lysine methylation enzymes [17]. All metazoans share a common set of cell signaling pathways [18], however the degree to which constituent proteins diverge in structure, copy number, or expression pattern varies across pathways [19]. Many signaling pathways are often characterized as “conserved”, not because individual protein sequences are constrained by natural selection, but because protein homologs occupy identical pathway positions across taxa and thus, presumably, perform similar functions. These results illustrate that while signaling pathways components may be “conserved”, that does not necessarily mean the protein sequences cease to produce adaptive mutations [20]. There are notable examples of natural selection co-opting developmental signaling pathways to produce evolutionary novelties and adaptations, however these usually involve changes to the pattern of expression, not structural mutations [21–23].

**Table 2:** The 11 genes experiencing recurrent positive natural selection

Gene	*n*_*c*_	*D*_*N*_	*D*_*S*_	*P*_*N*_	*P*_*S*_	*P*_FET_	Phenotype(s)
*nonC*	1852	31	39	8	53	0.000076	Hippo decrease
							Hedgehog increase
*Set2*	1408	27	37	17	73	0.001521	Hedgehog increase
*Nup153*	1294	49	49	8	30	0.001623	Hedgehog decrease
							RTK-Ras-ERK signaling decrease
							RTK-Ras-ERK signaling increase
*Kib*	1076	5	26	0	62	0.003269	Decreased cell viability
*pcm*	1072	29	28	4	19	0.005061	*Blood* TE activity
*Cnot4*	860	20	20	9	34	0.005227	Hippo decrease
*ZC3H3*	414	9	8	6	31	0.007610	Hippo increase
							RTK-Ras-ERK signaling decrease
*Nup205*	781	11	21	2	28	0.007613	Wnt increase
*Dref*	554	6	20	0	29	0.007942	Hedgehog increase
							RTK-Ras-ERK signaling decrease
*Sik3*	571	6	7	4	37	0.008072	Hippo decrease
*RasGAP1*	979	6	26	1	54	0.009106	Innate immunity
							RTK-Ras-ERK signaling increase

For each gene, the number of codons analyzed (*n*_*c*_), the number of nonsynonymous (*D*_*N*_) and synonymous (*D*_*S*_) substitutions, the number of nonsynonymous (*P*_*N*_) and synonymous (*P*_*S*_) polymorphisms are given along with the *P*-value for Fisher’s exact test (*P*_FET_) and the RNAi knockdown phenotypes affected by the gene

Although a handful of adaptively evolving proteins in signaling pathways are exclusive to just one phenotype, many proteins also play a role in multiple cell signaling pathways. For example, the *RasGap1* and *Dref* genes encode proteins with a history of recurrent positive selection and are involved in multiple signaling pathway phenotypes (Table 2). Both *RasGap1* and *Dref* play a role in Ras-mediated signal transduction [24, 25], which activates multiple downstream signaling pathways. Other positively selected proteins influencing cell signaling activity perform more general cellular functions. For instance, two nucleoporin genes (*Nup153*, *Nup205*) are both positively selected. While *Nup153* is involved in multiple signaling RNAi knockdown phenotypes, *Nup205* is identified as significantly influencing the Wnt signaling pathway. Nucleoporin genes encode components of the nuclear pore complex and therefore play a very general role in nuclear transport; these genes have previously been shown to be adaptively evolving in *D. melanogaster* [26]. Interestingly, one of the knockdown phenotypes influenced by *Nup153* is also influenced by the positively selected CCCH-type zinc finger gene *ZC3H3*; *ZC3H3* encodes a necessary component linking mRNA polyadenylation with nuclear export [27]. Both groups of proteins are known to interact with viral proteins [28, 29], which may be a potential source of selective pressure.

In addition to cell signaling pathways, our analysis identifies a new candidate for positively selected proteins in the piRNA pathway. The piRNA pathway generates small RNAs that suppress transposable element (TE) activity in the germline [30]. The piRNA effector proteins Mael, Armi, Aub, and Spn-E have been previously shown to experience positive natural selection in the *Drosophila* phylogeny [6]. Our analysis identifies a gene *pcm*, which both affects TE activity and shows an increased rate of adaptive amino acid substitutions in the *D. melanogaster* lineage (Table 2). *pcm* encodes a 5^′^−3^′^ exoribonuclease that has been previously characterized as having significant sequence conservation between *Drosophila*, mouse, and *Saccharomyces* [31]. The Pcm protein is recruited by protein complexes involved in both non-sense mediated mRNA decay (NMD) and RNA interference to degrade targeted mRNAs in cytoplasmic P-bodies [32].

### The effects of pleiotropy

**Fig. 1:**
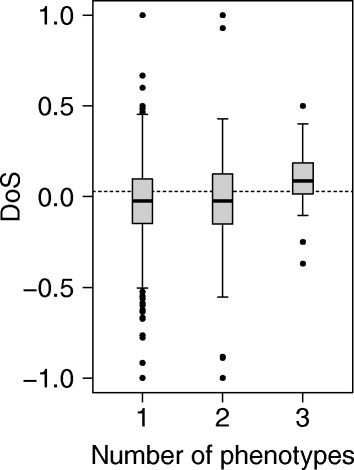
The number of phenotypic categories and the rate of protein evolution. The number of phenotypes for individual proteins is measured by the number of distinct categories of RNAi phenotypes that are significantly affected by knockdown of the corresponding gene. The box plots show the distribution of direction of selection (DoS) values for three different categories: genes that significantly affect one, two, and three different phenotypes upon RNAi knockdown. A positive DoS value indicate an excess of the proportion of substitutions in *D. melanogaster* that are nonsynonymous substitutions relative to the proportion of polymorphisms that are nonsynonymous. The mean DoS for genes involved in three categories of phenotype is significantly more positive compared to genes involved in either one or two phenotypic categories

Among the 11 genes that we infer to be subject to recurrent positive natural selection, four genes are also associated with multiple categories of RNAi knockdown phenotype (Table 2). Given that only seven of the 723 genes associated with a single category of RNAi knockdown phenotype show a history of adaptive evolution, observing four adaptively evolving genes involved in multiple categories is too many to occur by chance alone (*P*_FET_=0.0397). To explore the hypothesis that the number of RNAi phenotype categories is associated with the rate of amino acid substitution, we again use the direction of selection statistic (DoS). In total, there are 723 genes associated with a single category of RNAi knockdown phenotype, 85 genes involved with two categories, and 20 genes involved in three categories of phenotype. The mean DoS for genes associated with a single phenotypic category is −0.0302, while the mean for genes associated with two categories is −0.0248, and the mean for genes associated with three categories is 0.0936 (Fig. 1). The genes involved in three categories of RNAi knockdown phenotypes have significantly higher mean DoS than genes involved in either a single category (Mann-Whitney *U* test, *P*_*MWU*_=0.0058) or genes involved in two categories (*P*_*MWU*_=0.0382). Conversely, genes involved in one and two categories of RNAi knockdown phenotype do not have significantly different mean values of DoS (*P*_*MWU*_=0.7038). We note that this result is inconsistent with previous studies of yeast two-hybrid protein-protein interactions showing that highly interacting proteins tend to evolve more slowly [33], although more recent results are consistent with our finding [34]. This inconsistency may reflect the fact that yeast two-hybrid studies measure physical interactions among proteins, but not necessarily the number of biological processes influenced by a protein. We propose that our approach is a more accurate measure of a protein’s pleiotropic effects than are physical interaction data. Lastly, this result is not an artifact of longer genes having greater power to reject neutrality [35], since we observe no relationship between number of codons and degree of pleiotropy (*r*^2^=0.0001; *P*=0.926).

**Fig. 2:**
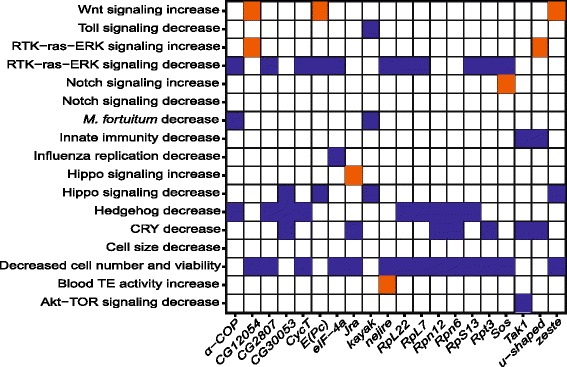
Cooperative or antagonistic pleiotropy for genes involved in three RNAi phenotypes. For the twenty highly pleiotropic genes, the tile plot shows the direction that gene knockdown has on a given phenotype. The blue tile shows negative effect on a phenotype upon knockdown; and the orange tile shows positive effect upon RNAi knockdown

We consider the number of distinct categories of RNAi knockdown phenotype as an indicator of the degree of protein pleiotropy. We find that the products of 20 genes have significant effects on three distinct categories of RNAi knockdown phenotype. We will refer to this group of genes as “highly pleiotropic”. Table 3 shows the combination of RNAi knockdown phenotypes, the number of nonsynonymous and synonymous substitutions and polymorphisms, as well as the DoS statistic for each of the highly pleiotropic genes. The most common set of phenotypic categories is “regulation of extent of cell growth”, coupled with “regulation of intracellular signal transduction” and “cell surface receptor signaling pathway”. Three of the eight genes associated with this combination of phenotypic categories encode ribosomal proteins (*RpL22*, *RpL7*, and *RpS13*). Across all twenty genes, there is no significant enrichment for gene ontology molecular function, the most enriched category is transcription factor binding (*u-shaped*, *kayak*, *nejire*, and *Jra*; *P*=0.090). Furthermore, of the 20 genes that significantly affect three different RNAi knockdown phenotypes, seven genes have opposite effects in at least two phenotypes upon knockdown. For example, *Sos* is a Ras-like guanine nucleotide exchange factor, which has a negative effect on the RTK-ras-ERK signaling pathway, whereas has a positive effect on the Notch signaling pathway upon knockdown (Fig. 2). Both in *C. elegans* and in *D. melanogaster*, Notch negatively regulates Ras pathway activation indicating antagonistic relationship between the Notch and the Ras signaling pathways [36]. Similarly, *u-shaped*, *E(Pc)*, and *zeste* have opposite effects in two different phenotypes upon RNAi knockdown. *u-shaped* is a Zinc-finger domain containing transcription factor, which upon knockdown, significantly increases the activity of the Ras signaling pathway, whereas has a negative effect on the immune deficiency (IMD) pathway that mediates innate immunity. The protein components of the Ras pathway are known to act as suppressors of the IMD pathway, even in the absence of immune challenges, indicating antagonistic relationship between the Ras and the IMD pathways [37]. Finally, two genes, *zeste* and *E(Pc)* have the same opposite effects for the Hippo pathway and the Wnt signaling pathway upon RNAi knockdown. Both *zeste* and *E(Pc)* increase the Wnt signaling activity, but influence Hippo signaling negatively upon knockdown. Similar to previous examples of antagonism between cellular pathways, the hippo pathway components are known to negatively regulate Wnt signaling genes [38]. While there are seven genes that have opposite effects in two pathways upon RNAi knockdown, 13 “highly pleiotropic” genes have similar effects on more than two phenotypes (Fig. 2). A majority of these genes (7 out of 13) significantly decrease Ras signaling, and Hedgehog signaling pathways upon knockdown. This result is consistent with the finding that both Ras and Hedgehog signaling pathways function cooperatively in cells [39, 40].

**Table 3:** Direction of selection (DoS) statistic for 20 genes involved in three different categories of RNAi knockdown phenotype

Gene	Phenotype categories^a^	*D*_*N*_	*D*_*S*_	*P*_*N*_	*P*_*S*_	DoS
*u-shaped*	CR-IM-SR	9	25	5	34	0.137
*kayak*	SR-IM-IS	5	11	5	26	0.151
*α* *COP*	SR-IM-IS	2	16	5	67	0.042
*E(Pc)*	SR-IM-IS	14	44	9	45	0.075
*Rpn6*	SR-CR-IS	0	6	1	15	–0.063
*CG30053*	SR-CR-IS	3	10	12	8	–0.369
*nejire*	GR-IS-TE	16	79	9	80	0.067
*Tak1*	GR-IM-IS	6	13	3	9	0.066
*eIF-4a*	GR-IM-IS	14	44	9	45	0.273
*Rpt3*	GR-CR-IS	2	7	0	16	0.222
*Jra*	GR-CR-IS	6	6	0	4	0.500
*CG12054*	GR-SR-IS	0	10	4	12	–0.250
*RpL22*	GR-SR-IS	1	6	0	3	0.143
*RpL7*	GR-SR-IS	3	6	0	8	0.333
*RpS13*	GR-SR-IS	2	3	0	1	0.400
*Sos*	GR-SR-IS	1	31	2	49	–0.008
*zeste*	GR-SR-IS	3	22	0	14	0.120
*CG2807*	GR-SR-IS	1	27	0	60	0.036
*CycT*	GR-SR-IS	5	29	5	15	–0.103
*Rpn12*	GR-SR-CR	1	9	0	1	0.100

^a^The two letter abbreviation codes for the phenotypic categories are: CR: Regulation of circadian rhythm, SR: Cell surface receptor signaling pathway, IS: Regulation of intracellular signal transduction, GR: Regulation of extent of cell growth, TE: Regulation of transposon integration, IM: Innate immune response

## Conclusions

*Drosophila melanogaster* represents one of most mature and powerful systems in genetics and functional genomics and is widely used as a model for studying the genetic basis of human disease [41, 42]. In particular, studies of *D. melanogaster* have led to significant advances in basic developmental, neurological, and immunological genetics. It is often stated that *D. melanogaster* is an appropriate genetic model because more than 60 % of the genes found in the *D. melanogaster* have human homologs [42] and that genes involved in key developmental pathways are “conserved” and functionally orthologous between humans and flies [41]. For *D. melanogaster* to be a viable human disease model, it is important to first understand the phenotypic effects of lineage-specific adaptations. While our results recapitulate the well-known conclusion that proteins affecting immunity and genome defense pathways are more likely to fix adaptive mutations, we also find that proteins affecting a suite of cell signaling pathways that are important for metazoan development are also fixing adaptive mutations in the *D. melanogaster* lineage at a significantly higher rate than the genome average. Our meta-analytical approach is conservative, such that we seek to minimize type I error in a potentially noisy data set. Less stringent criteria for statistical significance may, in fact, yield a different set of conclusions. However, our stringency adds to our confidence that the results do reflect the underlying biological realities concerning the molecular phenotypic effects of adaptive protein evolution.

In general, we refrain from speculating on the nature of the selective pressures driving the inferred adaptive evolution. However, it is important to note that the traditional MK framework used here is designed to detect recurrent bouts of adaptive evolution. One common explanation for recurrent positive selection is conflict due to an ongoing “arms race” between a host genome and either exogenous factors, such as pathogens [43], or endogenous selfish genetic elements, such as TEs or meiotic drive loci [44]. An “arms race” scenario would certainly apply to proteins involved in immunity or genome defense, as well as to proteins with general functions that interact with exogenous protein [45], such as is the case with the nucleoporins. Another potential source of recurrent positive selection is compensatory evolution [46]. Compensatory substitutions may resolve any antagonistic effects on fitness caused by an initial adaptive substitution. For instance, if strong positive selection fixes a mutation based on one aspect of the protein’s function, but that mutation also has lesser, deleterious effects on other aspects of the protein’s function, then natural selection will favor subsequent mutations that ameliorate these antagonistic effects. Our inference that proteins affecting a diverse range of molecular pathways are also more likely to experience adaptive evolution is consistent with this hypothesis. This conclusion lends support to two previous results that highlight the potential importance of compensatory evolution. The first is taken from evolutionary theory on the “cost of complexity”, which predicts that adaptive walks are characterized by initial mutations with large fitness effects, followed by mutations of smaller effect [47]. Empirical evidence also suggests that compensatory substitution is common: amino acid substitutions in *D. melanogaster* are observed to cluster according to their location in a protein’s tertiary structure [48], suggesting compensatory substitutions occur to preserve functional integrity. Because the MK-based framework is a widely used tool to infer the action of natural selection, the ability to distinguish “arms race” scenarios from compensatory evolution promises to bring unique new insights into the mode of protein evolution.

## Methods

### RNAi data

Data for 26 RNAi screens in *Drosophila melanogaster* are compiled from the GenomeRNAi database, release 3.0 [49]. All screens report standardized *Z* scores, which measure the effect that knocking down a single gene has on a phenotype, relative to that of a control gene. Across studies, we consider genes with *Z*<−3 or *Z*>3 to have significant effects on a phenotype. Positive and negative tails of *Z* are sampled depending on the phenotype, for example the negative tail of *Z* is taken for the JAK/STAT signaling decrease and the positive tail is taken for the JAK/STAT signaling increase. Off-target effects in RNAi screens may potentially overestimate the effects of single genes [50], all of the RNAi experiments cited in this study report designing dsRNA to be specific to single genes and, in some cases, knockdown effects are further validated by a variety of methods. Individual RNAi phenotypes are grouped into categories that reflect the deepest level of functional ontology that are shared by all of the phenotypes.

### Population genomic data

Reference-based genome assemblies of six European and nine sub-Saharan African strains of *D. melanogaster* (Additional file 1: Table S1) are generated from short-read data in the NCBI short read archive [51]. Reads are mapped to the genome of the reference *D. melanogaster* strain *y*^1^;*cn*^1^*bw*^1^*sp*^1^ (version 5.45) using the BWA software [52]. Variants are called using the POPBAM software with default settings [53]. Gene alignments are then constructed for the longest transcript per gene from the FlyBase mRNA annotations, using the Perl script PBsnp2fa.pl (https://github.com/skingan/PBsnp2fa.pl). A total of 13329 alignments were initially constructed. Ancestral and derived states are inferred by aligning to the genomes of both *D. simulans* strain MD063 [54] and *D. yakuba* strain Tai18E2 [55]. Requiring sequence alignment to both *D. simulans* and *D. yakuba* limits the data set to 11148 total gene alignments (2839 gene alignments are dropped) and it is likely that very rapidly evolving genes may not appear in the final data set.

### Tests of natural selection

To determine the relative effects of natural selection across different RNAi knockdown phenotypes, we perform two analyses. First, we ask whether the genes associated with each RNAi knockdown phenotype, as a group, are enriched for amino acid substitutions (indicating adaptive evolution). We ask whether the genes significantly affecting each phenotype have an increased number of nonsynonymous substitutions compared to nonsynonymous polymorphisms using the direction of selection (DoS) statistic [9]. The DoS statistic is defined as the difference between the proportion of nonsynonymous substitutions (*D*_*N*_) to the sum of synonymous substitutions (*D*_*S*_) and nonsynonymous substitutions (*D*_*N*_) and the proportion of nonsynonymous polymorphisms (*P*_*S*_) to the sum of synonymous polymorphisms (*P*_*S*_) and nonsynonymous polymorphisms (*P*_*N*_), given as DoS=*D*_*N*_/(*D*_*S*_+*D*_*N*_)−*P*_*N*_/(*P*_*S*_+*P*_*N*_). Statistical significance of DoS is assessed by a bootstrap procedure, in which a null distribution is calculated by selecting a random sample of *N* genes from the genome, where *N* is the number of genes in the phenotype to be evaluated. Significance is assessed for DoS using a two-tailed approach, therefore empirical values are considered significant if they fall outside 0.975 quantile of the null distribution. For each phenotype, 10000 bootstrap replicates are performed using the statistical programming language R.

Our second analysis uses single locus MK tests to evaluate individual gene alignments for signatures of positive natural selection. The MK test considers the null hypothesis that the ratio of nonsynonymous (*D*_*N*_) and synonymous (*D*_*S*_) substitutions between *D. melanogaster* and *D. simulans* is equal to the ratio of nonsynonymous (*P*_*N*_) and synonymous (*P*_*S*_) polymorphisms within *D. melanogaster* [56]. Given that the ratio of *P*_*N*_/*P*_*S*_ forms the expectation for the ratio of *D*_*N*_/*D*_*S*_, we calculate probability of obtaining *D*_*N*_ higher than the observed value using a one-tailed Fisher’s exact test (FET). Gene alignments with fewer than six sites in any of the marginal counts are considered to have zero power [35]. Of the original 11148 MK tests, by the above criterion, 4063 tests are determined to have zero power and are subsequently removed [57] leaving 7085 valid tests. It is likely that removing low-power tests results in the elimination of genes experiencing a low rate of neutral mutation, but since there is no predictable relationship between neutral mutation rate and the distribution of fitness effects [58], there is no concrete *a priori* reason to believe this procedure will systematically bias our analysis of natural selection. From the remaining valid MK tests, using the Perl script mk-fdr.pl (https://github.com/dgarriga/mk-fdr) the proportion that are truly null is estimated to be 0.978, using a method designed to analyze *P*-value distributions from conservative tests [54]. At the 5 % level of significance, this corresponds to a false discovery rate of 40.9 %. However, we only consider tests with *P*_FET_<0.01 to be statistically significant, which corresponds to a false discovery rate of 22.7 %. Finally, it should be noted that we observe a significant negative correlation between the number of codons in a gene and the MK test *P*-value (*r*^2^=0.01334; *P*≪0.001).

## Additional file

Additional file 1
**Supplementary Tables.** **Table S1.** The 26 RNAi knockdown phenotypes surveyed in this study. We identify phenotypes that are enriched for proteins that significantly deviate from the genome average in their direction of selection (DoS). **Table S2.** Details of the 15 *Drosophila melanogaster* strains used in this study, including database accession numbers and identifiers [51], percent of the reference genome with reads mapped from that strain, and the average read depth across the entire genome assembly. (PDF 103 kb)
