# Supplementary material for: The effects of natural selection across molecular pathways in Drosophila melanogaster
Source: BMC Evol Biol. 2015 Sep 21;15:203. doi: 10.1186/s12862-015-0472-4 (PMC4578789; doi:10.1186/s12862-015-0472-4)
Supplement: Additional file 1 — Supplementary Tables. Table S1. The 26 RNAi knockdown phenotypes surveyed in this study. We identify phenotypes that are enriched for proteins that significantly deviate from the genome average in their direction of selection (DoS). Table S2. Details of the 15 Drosophila melanogaster strains used in this study, including database accession numbers and identifiers [51], percent of the reference genome with reads mapped from that strain, and the average read depth across the entire genome assembly. (PDF 103 kb) [file 12862_2015_472_MOESM1_ESM.pdf]

RESEARCH

Supplementary Material: The effects of natural selection across molecular pathways in *Drosophila melanogaster*

Jeffrey P. Vedanayagam[†] and Daniel Garrigan[*]

[*]Correspondence:
dgarriga@ur.rochester.edu
Department of Biology, University of Rochester, 14627 Rochester, New York, USA
Full list of author information is available at the end of the article
[†]jvedanay@ur.rochester.edu

Table 1 The 26 RNAi knockdown phenotypes surveyed in this study. We identify phenotypes that are enriched for proteins that significantly deviate from the genome average in their direction of selection (DoS).

RNAi knockdown phenotype	RNAi ID	Ontology	Total[1]	n^2	Reference
Whole-genome	–	–	–	11148	–
Lethals	–	–	–	2853	–
Akt-TOR signaling decrease	GR00169	GO:1902531	13900	24	[1]
Akt-TOR signaling increase	GR00169	GO:1902531	13900	22	[1]
Blood TE activity increase	GR00284	GO:0070894	8171	43	[2]
Burdock TE activity increase	GR00284	GO:0070894	8171	15	[2]
Cell size regulation	GR00001	GO:0061387	13900	5	[3]
CRY degradation	GR00006	GO:0042752	13900	93	[4]
Decreased cell number and viability	GR00031	GO:0061387	13071	96	[5]
Hedgehog signaling decrease	GR00045	GO:0007166	5828	48	[6]
Hedgehog signaling increase	GR00045	GO:0007166	5828	30	[6]
HeTA TE activity increase	GR00284	GO:0070894	8171	38	[2]
Hippo signaling decrease	GR00218	GO:1902531	13059	99	[7]
Hippo signaling increase	GR00218	GO:1902531	13059	54	[7]
Hypoxia induced transcription	GR00170	GO:0097411	13071	49	[8]
Influenza replication decrease	GR00086	GO:0045087	13071	18	[9]
Innate immunity	GR00041	GO:0045087	13071	13	[10]
JAK/STAT signaling decrease	GR00044	GO:1902531	12649	10	[11]
JAK/STAT signaling increase	GR00044	GO:1902531	12649	6	[11]
M. fortuitum infection decrease	GR00046	GO:0045087	12740	27	[12]
Notch signaling decrease	GR00152	GO:0007166	13900	16	[13]
Notch signaling increase	GR00152	GO:0007166	13900	18	[13]
RTK-Ras-ERK signaling decrease	GR00214	GO:1902531	13900	113	[14]
RTK-Ras-ERK signaling increase	GR00214	GO:1902531	13900	55	[14]
TAHRE TE activity increase	GR00284	GO:0070894	8171	41	[2]
Toll signaling decrease	GR00176	GO:0007166	13900	17	[15]
Toll signaling increase	GR00176	GO:0007166	13900	11	[15]
Wnt signaling activity	GR00040	GO:0007166	13071	90	[16]

[1] Total number of gene knockdowns assayed for effects on a given phenotype
[2] Number of genes significantly affecting the RNAi knockdown phenotype

Table 2 Details of the 15 Drosophila melanogaster strains used in this study, including database accession numbers and identifiers [17], percent of the reference genome with reads mapped from that strain, and the average read depth across the entire genome assembly.

SRA accession	DPGP stock ID	Origin	% coverage	Read depth
SRX058182	FR14	France	96.12	34.36
SRX058183	FR151	France	96.63	34.95
SRX058185	FR207	France	97.73	34.84
SRX058186	FR217	France	96.73	35.66
SRX058188	FR310	France	97.67	33.78
SRX058189	FR361	France	96.56	35.89
SRX058338	RG10	Rwanda	96.88	34.30
SRX058339	RG11N	Rwanda	98.46	33.32
SRX058182	RG15	Rwanda	97.82	41.73
SRX058346	RG21N	Rwanda	94.72	32.88
SRX058352	RG32N	Rwanda	97.85	34.88
SRX058354	RG34	Rwanda	98.03	28.75
SRX058355	RG35	Rwanda	97.81	30.90
SRX058357	RG36	Rwanda	98.11	28.75
SRX058359	RG38N	Rwanda	96.26	34.05

References

1. Kockel, L., Kerr, K.S., Melnick, M., Bruckner, K., Hebrok, M., Perrimon, N.: Dynamic switch of negative feedback regulation in *Drosophila* Akt-TOR signaling. PLoS Genet **6**, 1000990 (2010)
2. Czech, B., Preall, J.B., McGinn, J., Hannon, G.J.: A transcriptome-wide RNAi screen in the *Drosophila* ovary reveals factors of the germline piRNA pathway. Mol Cell **50**, 749–761 (2013)
3. Bjorklund, M., Taipale, M., Varjosalo, M., Saharinen, J., Lahdenpera, J., Taipale, J.: Identification of pathways regulating cell size and cell-cycle progression by RNAi. Nature **439**, 1009–1013 (2006)
4. Sathyanarayanan, S., Zheng, X.Z., Kumar, S., Chen, C.H., Chen, D.C., Hay, B., Sehgal, A.: Identification of novel genes involved in light-dependent CRY degradation through a genome-wide RNAi screen. Genes Dev **22**, 1522–1533 (2008)
5. Boutros, M., Kiger, A.A., Armknecht, S., Kerr, K., Hild, M., Koch, B., Haas, S.A., Paro, R., Perrimon, N., Consortium, H.F.A.: Genome-wide RNAi analysis of growth and viability in *Drosophila* cells. Science **303**, 832–835 (2004)
6. Lum, L., Yao, S.Q., Mozer, B., Rovescalli, A., Von Kessler, D., Nirenberg, M., Beachy, P.A.: Identification of hedgehog pathway components by RNAi in *Drosophila* cultured cells. Science **299**, 2039–2045 (2003)
7. Wehr, M.C., Holder, M.V., Gailite, I., Saunders, R.E., Maile, T.M., Ciirdaeva, E., Instrell, R., Jiang, M., Howell, M., Rossner, M.J., Tapon, N.: Salt-inducible kinases regulate growth through the Hippo signalling pathway in *Drosophila*. Nat Cell Biol **15**, 61–71 (2013)
8. Dekanty, A., Romero, N.M., Bertolin, A.P., Thomas, M.G., Leishman, C.C., Perez-Perri, J.I., Boccaccio, G.L., Wappner, P.: *Drosophila* genome-wide RNAi screen identifies multiple regulators of HIFdependent transcription in hypoxia. PLoS Genet **6**, 1000994 (2010)
9. Hao, L.H., Sakurai, A., Watanabe, T., Sorensen, E., Nidom, C.A., Newton, M.A., Ahlquist, P., Kawaoka, Y.: *Drosophila* RNAi screen identifies host genes important for influenza virus replication. Nature **454**, 890–893 (2008)
10. Foley, E., O'Farrell, P.H.: Functional dissection of an innate immune response by a genome-wide RNAi screen. PLoS Biol **2**, 203 (2004)
11. Muller, P., Kuttenkeuler, D., Gesellchen, V., Zeidler, M.P., Boutros, M.: Identification of JAK/STAT signalling components by genome-wide RNA interference. Nature **436**, 871–875 (2005)
12. Philips, J.A., Rubin, E.J., Perrimon, N.: *Drosophila* RNAi screen reveals CD36 family member required for mycobacterial infection. Science **309**, 1251–1253 (2005)
13. Mourikis, P., Lake, R.J., Firnhaber, C.B., DeDecker, B.S.: Modifiers of notch transcriptional activity identified by genome-wide RNAi. BMC Dev Biol **10**, 107 (2010)
14. Friedman, A.A., George, T., Rohit, S., Dong, Y., Arunachalam, V., Yanhui, H., Richard, B., Pengyu, H., Xiaoyun, S., Maura, P., Svetlana, P., Thilakam, M., L., F.R., M., A.J., Bonnie, B., Norbert, P.: Proteomic and functional genomic landscape of receptor tyrosine kinase and Ras to extracellular signalregulated kinase signaling. Sci Signal **4**, 10 (2011)
15. Kuttenkeuler, D., Pelte, N., Ragab, A., Gesellchen, V., Schneider, L., Blass, C., Axelsson, E., Huber, W., Boutros, M.: A large-scale RNAi screen identifies Deaf1 as a regulator of innate immune responses in *Drosophila*. J Innate Immun **2**, 181–194 (2010)
16. DasGupta, R., Nybakken, K., Booker, M., Mathey-Prevot, B., Gonsalves, F., Changkakoty, B., Perrimon, N.: A case study of the reproducibility of transcriptional reporter cell-based RNAi screens in *Drosophila*. Genome Biol **8**, 203 (2007)
17. Pool, J., Corbett-Detig, R., Sugino, R., Stevens, K., Cardeno, C., Crepeau, M., Duchen, P., Emerson, J., Saelao, P., Begun, D., Langley, C.: Population genomics of sub-Saharan *Drosophila melanogaster*: African diversity and non-African admixture. PLoS Genet **8**, 1003080 (2012)
